# The EPS Matrix as an Adaptive Bastion for Biofilms: Introduction to Special Issue

**DOI:** 10.3390/ijms141223297

**Published:** 2013-11-26

**Authors:** Alan W. Decho

**Affiliations:** Microbial Interactions Laboratory, Department of Environmental Health Sciences, Arnold School of Public Health, University of South Carolina, Columbia, SC 29208, USA; E-Mail: awdecho@mailbox.sc.edu; Tel.: +1-803-777-6584; Fax: +1-803-777-3391

The process of biofilm formation has knowingly, and even unsuspectingly, baffled scientists for almost as long as the field of microbiology itself has existed. This Special Issue of the International Journal of Molecular Sciences (IJMS) specifically addresses an important component of the biofilm, the extracellular matrix. This matrix forms the protective secretions that surround biofilm cells and afford a “built environment” to contain biofilm processes. During the earlier days of microbiology, it was intriguing to Claude ZoBell that attached bacteria sometimes were able to proliferate when their planktonic counterparts were unable to grow [1]. During the 1970s, this attached state was beginning to be explored [2], and it was realized to be anchored in a matrix of slime-like molecules. The slime-like matrix together with cells was to be called the “biofilm”, a term developed by the late Bill Costerton, Bill Characklis and colleagues. The scientific revelation that attached bacteria were different from free (*i.e.*, planktonic) cells in their physiological behavior and adaptability, launched an era of focused exploration in this area of microbiology. It was initially surprising, though not unexpected in retrospect, that interest in biofilms has grown and now infiltrates virtually all aspects of our scientific study. Since that time there has been a near-exponential growth in the numbers of scientific publications addressing biofilms owing to their immediate relevance to ecology, biotechnology, health and industry.

During this exciting time, it was shown that the same strains of bacteria, when grown as free-cells (*i.e.*, plankton) *vs*. attached cells (*i.e*., biofilm), exhibited differences in gene expression, cell–cell chemical communication, microspatial distributions, enzyme activities, antibiotic production, physical resistance to dispersion under flow, and other interactions between cells within biofilms [3,4]. However, one conspicuous gap emerged in understanding the biofilm. While there was much focus on biofilm cells, there was a relative paucity of studies addressing the extracellular matrix just outside the microbial cell boundaries, even though the extracellular matrix was recognized to be an integral part of the biofilm. It is the extracellular matrix that provides the physical architecture for interactions and facilitates feedback (sensing and signaling) among cells; two essential properties, which allow attached cells to operate differently from their planktonic counterparts.

During the 1990s, the acronym “*EPS*” was developed by Thomas Neu, Hans-Curt Flemming and colleagues, to encompass the extracellular polymeric substances or secretions. EPS was coined to emphasize the wide range of molecules such as proteins, polysaccharides, nucleic acids, and lipids, which comprise these secretions. It is a primary emergent property of the biofilm. Many in-depth reviews and overviews have addressed the specific topic of EPS, and have begun to reveal its complexity, and the difficulty in determining how this extracellular biome influences cells [5–9]. These reviews serve as a foundation for understanding the current knowledge and significant gaps in EPS-related research.

The biofilm, and specifically its EPS-related architecture, are now recognized as an important contributor to the areas of health and disease [10,11], industrial biofouling, biotechnology, and more-general ecosystem health. In the area of microbial ecology, EPS provides the physical architecture to support the incredible diversities observed in microbial mats and natural surface microbial communities, though its roles in this support are poorly understood. Complex communities such as those of microbial mats consist of tens of thousands of microbial species, as determined by 16S rDNA sequencing. The complex communities are tightly enclosed within largely-uncharacterized forms of environmentally-modified EPS, which have evolved and successfully adapted mat systems for literally billions of years. EPS is closely-linked to biogeomineral precipitation [12], and is used to interpret the microbial fossil record and remnants of the earliest life on Earth [13]. It is also being studied in the development of “microbial cement” used to repair cracks in building, statues and engineered structures. Periodontal disease, and its microbial cells anchored in EPS, is now realized to be a complex interplay between hundreds of commensal bacterial species that coexist within distinctly different environments of the oral cavity, and invasive forms reaching beyond those boundaries into the body [14,15]. The human gut microbiome is a recent emerging area of focus in health, and is now realized to be largely biofilm-based [16].

EPS has been difficult to characterize and to define beyond their bulk properties. This is largely because EPS molecules, their interactions, and their multi-functional roles to cells are diverse, and do not lend themselves to standard predictability, nor analyses by standard molecular tools. However, new tools are emerging, or rather, becoming more user-friendly, for the microbiologist and can be used to investigate such small-scale interactions within biofilms, and specifically the diverse EPS components. It is now realized that the study of EPS needs to be addressed (ideally) with minimal disturbance to the matrix during analyses. New approaches can now achieve nanometer-, even angstrom-level spatial resolution of molecules, which are necessary to begin understanding the complexity of EPS. It is especially important to probe the matrix *in situ*, wherever possible, in order to understand the “functional units” of EPS. That is, how do groups of molecules interact and result in a “function(s)” to cells. Understanding these interactions will reduce to deciphering the basic physical chemistry, while ascertaining their biological role(s). The many interactions occurring among the microbial flora of the gut, such as chemical communication and sensing, are related to EPS-localized processes. Biofilm microorganisms and the unique properties of their EPS are now being explored and exploited in biotechnology for food additives, as well as in pharmaceuticals, for drug delivery.

This Special Issue is not intended to be all-encompassing in its coverage of EPS, but rather to present a series of EPS studies, and reviews, from different areas of investigation ranging from the environment to health.

## References

[1] ZoBell C.E. (1943). The effect of solid surfaces on bacterial activity. J. Bacteriol.

[2] Costerton J.W., Geesey G.G., Chang K.-J. (1978). How bacteria stick. Sci. Am.

[3] Davies D.G., Parsek M.R., Pearson J.P., Iglewski B.H., Costerton J.W., Greenberg E.P. (1998). The involvement of cell-to-cell signals in the development of a bacterial biofilm. Science.

[4] Stoodley P., Cargo R., Rupp C.J., Wilson S., Klapper I. (2003). Biofilm material properties as related to shear-induced deformation and detachment phenomena. J. Ind. Microbiol. Biotechnol.

[5] Neu T.R., Lawrence J.R., Wingender J., Neu T.R., Flemming H.-C. (1999). *In situ* Characterization of Extracellular Polymeric Substances (EPS) in Biofilm Systems. Microbial Extracellular Polymeric Substances.

[6] Flemming H.C., Neu T.R., Wozniak D. (2007). The EPS matrix: The house of biofilm cells. J. Bacteriol.

[7] Böckelmann U., Janke A., Kuhn R., Neu T.R., Wecke J., Lawrence J.R., Szewzyk U. (2006). Bacterial extracellular DNA forming a defined network-like structure. FEMS Microbiol. Lett.

[8] Flemming H.-C., Wingender J. (2010). The biofilm matrix. Nat. Revs. Microbiol.

[9] Decho A.W. (1990). Microbial exopolymer secretions in ocean environments: Their roles in food webs and marine processes. Oceanogr. Mar. Biol. Ann. Rev.

[10] Costerton J.W., Stewart P.S., Greenberg E.P. (1999). Bacterial biofilms: A common cause of persistent infections. Science.

[11] Hall-Stoodley L., Stoodley P. (2005). Bacterial biofilms: From the natural environment to infectious diseases. Nat. Rev. Microbiol.

[12] Dupraz C., Reid R.P., Braissant O., Decho A.W., Norman R.S., Visscher P.T. (2010). Processes of carbonate precipitation in modern microbial mats. Earth Sci. Rev.

[13] Nofke N., Decho A.W., Stoodley P. (2013). Slime through time: The fossil record of prokaryote evolution. Palaios.

[14] Jenkinson H.F., Lamont R.J. (2005). Oral microbial communities in sickness and in health. Trends Microbiol.

[15] Kuramitsu H.K., He X., Lux R., Anderson M.H., Shi W. (2007). Interspecies interactions within oral microbial biofilms. Microbiol. Mol. Biol. Rev.

[16] Bäckhed F., Ley R.E., Sonneburg J.L., Peterson D.A., Gordon J.I. (2005). Host-bacterial mutualism in the human intestine. Science.

